# A79 AIMING TO PROVIDE EARLIER CARE WHILE REDUCING SPECIALTY CLINIC WAIT TIMES: DEVELOPING A RELIABLE PROCESS OF ASSESSING THE ELIGIBILITY OF GASTROENTEROLOGY REFERRALS FOR PRIMARY CARE PATHWAYS

**DOI:** 10.1093/jcag/gwab049.078

**Published:** 2022-02-21

**Authors:** D Budhram, E Neary, M Kelley, L Hookey

**Affiliations:** 1 Queen’s University, Kingston, ON, Canada; 2 Medicine, Queen’s University, Kingston, ON, Canada; 3 Gastrointestinal Diseases Research Unit, Queen’s University, Kingston, ON, Canada

## Abstract

**Background:**

Gastrointestinal (GI) diseases are common and are a source of substantial morbidity, mortality, and cost, accounting for the largest share of medical referrals from primary care physicians (PCPs) in Canada. Long wait times and increasing numbers of referrals for GI consultation have thus become a significant issue, and while awaiting consultation many patients experience impaired quality of life. Primary care clinical pathways are structured care plans outlining detailed steps for treating patients with specific GI conditions, supporting the translation of clinical guidelines into local protocols and clinical practice.

**Aims:**

To identify the proportion of referrals sent from PCPs to the Queen’s Gastroenterology division that meet the eligibility criteria for a clinical care pathway.

**Methods:**

A review was conducted to identify the proportion of non-urgent referrals in triage sent from PCPs to the Queen’s Gastroenterology division that met the criteria for a clinical care pathway from July 2019 to May 2020. The reason for referral from PCP to gastroenterology was recorded. Individual patient characteristics included in each referral letter were assessed, including age, sex, GI symptoms or signs, relevant clinical features (i.e. time course of symptoms, frequency of bowel movements), relevant laboratory parameters (i.e. CBC, LFTs, electrolytes) or any investigations ordered to evaluate the cause of the patient’s symptoms or signs by the PCP. Four reviewers assessed an initial sample of 200 referral letters and classified each patient’s eligibility for a primary care pathway (GERD, diarrhea, IBS, dyspepsia, or ineligible). After every 100 referrals, discrepancies were reviewed, and a consensus was reached for each case. Data from 100 subsequent referral letters were collected by two independent reviewers, and the inter-rater reliability was calculated. Following a high level of agreement, the final 70 referrals were assessed by two reviewers.

**Results:**

Out of 370 referrals sent from PCPs to the Queen’s Gastroenterology division from July 2019 to December 2019, a total of 170 (46%) met the eligibility criteria for a clinical care pathway. From the eligible referrals, the proportion of patients in each pathway were as follows: 35% GERD, 12% diarrhea, 34% IBS and 19% dyspepsia. The inter-rater reliability for the first 100 referrals assessed by two independent reviewers was 94% (κ = 0.873).

**Conclusions:**

These findings demonstrate that a significant proportion of the patient population referred from PCPs to Queen’s Gastroenterology would be eligible for a primary care pathway. Future steps include implementing these pathways to determine their effectiveness in reducing wait times and empowering PCPs to care for patients who do not require specialist referral.

**Funding Agencies:**

None

